# Leaf nitrogen and phosphorus stoichiometry of the halophytes across China

**DOI:** 10.3389/fpls.2023.1276699

**Published:** 2023-10-04

**Authors:** Ran Tong, Cong Ma, Chenyang Lou, Wenwen Yuan, Nianfu Zhu, G. Geoff Wang, Tonggui Wu

**Affiliations:** ^1^ East China Coastal Forest Ecosystem Long-term Research Station, Research Institute of Subtropical Forestry, Chinese Academy of Forestry, Hangzhou, China; ^2^ Department of Forestry and Environmental Conservation, Clemson University, Clemson, SC, United States

**Keywords:** ecosystem type, growth form, halophyte, scaling relationship, stoichiometry

## Abstract

Halophytes play a crucial role in the ecological restoration of saline and alkaline land and hold promising benefits to food security in China. Although a variety of aspects of halophytes have been extensively addressed, there is still a lack of overall understanding of the leaf nitrogen (N) and phosphorus (P) stoichiometric characteristics, especially at a national scale. We compiled a national dataset of 311 observations from 113 sampling sites across China to explore the changing trends and influencing factors on leaf N and P concentrations, and N:P ratio of halophytes. The results showed that leaf N concentration decreased significantly with increasing latitude (LAT), which was mainly driven by the mean annual temperature (MAT) and mean annual precipitation (MAP). The leaf P concentration increased remarkably with increasing longitude (LON), which was induced by the variation in soil total P (TP) content. The leaf N:P ratio increased as LAT increased and LON decreased, which was potentially regulated by the MAT, MAP, and soil TP content. The scaling exponents of the N-P relationship differed significantly among halophyte types and were 0.40, 0.87, and 1.39 for euhalophyte, pseudohalophyte, and recretohalophyte, respectively. The leaf N concentration exhibited significant differences among ecosystem types and halophyte types, whereas the leaf P concentration and N:P ratio remained relatively stable. In summary, the leaf N concentration and N-P scaling exponent might be the classification criteria for halophyte types from the perspective of plant nutrient resource allocation. Moreover, this study characterized the spatial distribution and allocation strategy of leaf N and P stoichiometry in halophytes by data integration analysis, providing the basic information for nutrient management in the processes of the future domestication and introduction of halophytes.

## 1 Introduction

The formation of plant flora is a comprehensive reflection of the evolution and spatial-temporal distribution in a particular historical and biogeographical environment (Chen et al., 2018); thus, nutrient traits can effectively express the mutual relations between plants and an environment in comparison to the individual species or multispecies collection. Research on breeding, physiology, and ecology research for natural plant flora has provided important scientific and technological support for the cultivation and breeding of agronomic crops, obtaining excellent economic benefits (van Kleunen et al., 2020). Moreover, plants from the habitats with certain species-specific particularities play a pivotal role in coping with global climate change, maintaining ecological balance, and protecting biodiversity (Shang et al., 2020; Liu and Wang, 2021).

Due to continued environmental deterioration induced by global changes, increased soil salinization has become a severe national problem in China (Xiao et al., 2019; Zhang et al., 2020). Currently, the saline-alkali soil in China covers approximately 99.1 million hectares, accounting for approximately 10% of the global saline-alkali land resources, thus seriously affecting the improvement of agricultural production and its efficiency in the country. Halophytes are a group of salt-tolerant plants that survive and generate high biomass in a saline soil environment, and they play an irreplaceable role in maintaining ecological balance and species diversity, as well as the improvement and utilization of saline and alkali soil (Zhao et al., 2002a; Zilaie et al., 2022). Therefore, the understandings of the ecological adaptability of halophytes under environmental changes probably provide guidelines for the cultivation of salt-tolerant grain crops and forage grasses, and further contribute to the improvement and utilization of saline-alkali land (Ruan et al., 2010; Ventura et al., 2015).

Since recognizing the importance of the halophytic resources, the current taxonomic status and distribution of halophytes have been systematically explored at home and abroad (Zhao et al., 2002b; Al Hassan et al., 2017; Wang et al., 2020). According to their geographical distribution characteristics, halophytes could be roughly divided into three types, including coastal land, grassland, and desert. The included plant species based on the geographical divisions have some specificity, whereas there are also widely distributed species included *Phragmites australis, Kalidium foliatum*, and *Suaeda* plants. In addition, based on their adaptation mechanisms to the high-salt environment, the halophytes could also be divided into euhalophytes, pseudohalophytes, and recretohalophytes physiologically (Mader et al., 2022; Meena et al., 2023). It should be noted that the different responses of halophytes and non-halophytes to salinity are considered to be expressed quantitatively (or by degree) rather than qualitatively (Flowers et al., 1986; Zhao, 1997). Therefore, it is of great significance to research the differences of leaf nutrient traits between halophytes and other plant groups, might providing new insights into the plant ecological adaptation to environmental change during long-term evolution.

Ecological stoichiometry (ES) is an elementary approach in which basic principles are used to explore the coupling of global N, and P cycles via their biotic interactions and their responses to climate change (Hessen et al., 2013). The identifications of the leaf N and P stoichiometric characteristics along various environmental gradients are considered to contribute to a deep understanding of the mechanisms involved in the response of plant traits and ecological adaptation to environmental changes, and further developing several theoretical frameworks and hypotheses (Zhang et al., 2018; Tian et al., 2019). For instance, the two leading hypotheses, temperature-plant physiological hypothesis (TPPH) and soil substrate age hypothesis (SAH), were presented to characterize the spatial patterns of leaf N and P stoichiometry and the response to environmental factors (Tian et al., 2021). In detail, the TPPH states that the accumulations of N and P concentration in leaves could compensate for the declines in metabolic rate at low temperature, and the SAH predicts that P-limitation of plant growth increases gradually from arctic to tropical regions and is mainly restricted by less phosphate rock weathering. Nevertheless, whether these prior theories are equally applicable to plant flora has not yet been verified. Although the spatial patterns of leaf N and P stoichiometry in individual species or multispecies collections have been well studied, few researchers have explored particular plant flora such as halophytes, especially at regional or larger scales.

China is a geographically diverse country, with many types of climate zones and various types of plant flora, making it an ideal place to explore the influence of environmental conditions on plant nutrient cycling and balance (Chen et al., 2015). In recent years, with the appearance of new thoughts regarding the utilization of halophytes, abundant halophytic resources have played a remarkable role in improving in the environmental quality of national saline-alkali lands and in the implementation of the “grain storage in land and technology” strategy in the new era (Wang et al., 2013; Zhao et al., 2013; Ventura et al., 2015).

In the present study, we collected data on leaf N and P stoichiometry, along with geographical, climatic, and soil nutrient information from published literature. Our main aims were to: 1) characterize the geographical patterns of leaf N and P stoichiometry; 2) identify the variation in leaf N and P stoichiometry among different ecosystems and plant types; 3) test whether the allometric scaling relationship exist among different halophyte types. Overall, our study was expected to enhance the understanding of the ecological adaptions of the halophytes and provide some basic information for the rational utilization of halophyte resources in the future.

## 2 Materials and methods

### 2.1 Dataset collection

The data were collected from the Web of Science and China National Knowledge Infrastructure (CNKI) database by using the keyword combinations, i.e., (halophyte OR salt-tolerant plant OR desalination plant) AND (plant OR leaf) AND (carbon OR nutrient OR nitrogen OR phosphorus OR stoichiometry). Data were collected from published studies mainly focused on the leaf N and P concentrations of halophytes, along with the geographical, climatic, and soil nutrient information associated with the leaf samples obtained from 2010 to 2021, mainly according to the saline soil characteristics of the halophyte distribution area in China. Moreover, authoritative texts of plant taxonomy were used to make sure the division accuracy of halophyte types for the included plant species (Zhao, 1999; Hao et al., 2006).

**Figure 1:**
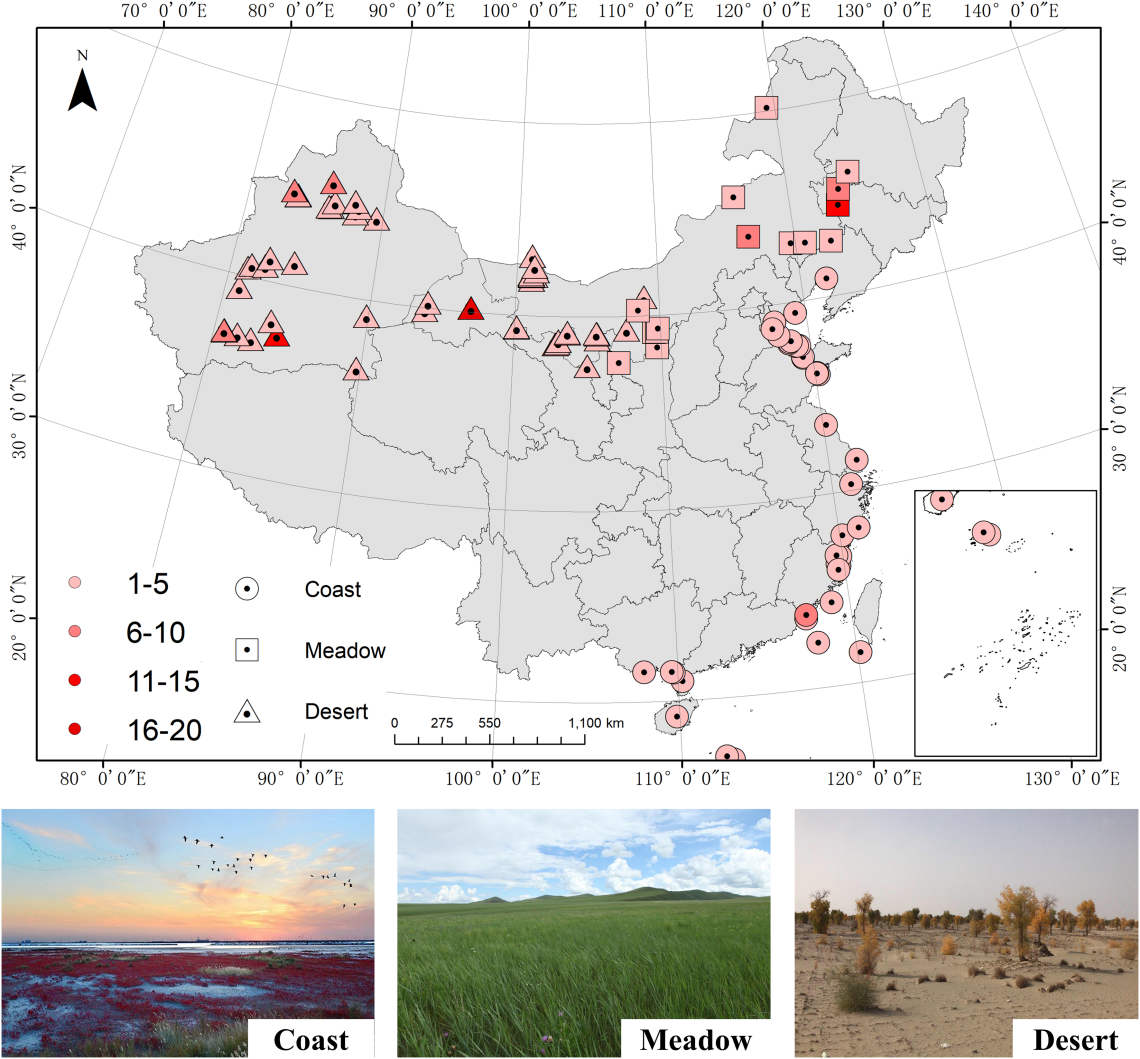
Distribution of sampling sites included in this integration analysis. The location types are shown in different shapes. Centre of shape: the locations of each site; shape color: number of observations at each site.

The database consisted of 311 observations from 113 sampling sites across China (**Figure 1**), including 74 halophytes in 57 genera and 28 families. For each sampling site, we also collected data on geographic, climatic, and soil nutrient content variables, such as the latitude (LAT, °), longitude (LON, °), altitude (ALT, m), mean annual temperature (MAT, °C), mean annual precipitation (MAP, mm), and soil total N (TN, g/kg) and soil total P (TP, g/kg) contents in the surface soil (mainly concentrated in the 0-30 cm soil layer) (**Appendix Table S1**). In addition, the data on soil pH and soil salinity (mg/g) was collected, and its correlations with leaf N and P stoichiometry were also examined (**Figure S1**). When studies did not provide the mean MAT or MAP, we obtained these data from the WordClim database [v.2.0; http://www.worldclim.org/; Fick and Hijmans (2017)] at a spatial resolution of 30″ using the corresponding coordinates.

The functional groups were also recorded, including the ecosystem type (desert, meadow, and coast), life form (herb, shrub, and tree), phylogenetic development (dicotyledon and monocotyledon), halophyte type (euhalophyte, recretohalophyte, and pseudohalophyte) and photosynthetic pathway (C3 and C4 herbs). Leaf samples, used for determining the leaf N and P concentrations, were collected during the growing season (mainly from July to October). The leaf N:P ratios were expressed on a mass basis.

Previously, Han et al. (2005) reported leaf N and P stoichiometry results across 753 terrestrial plant species in China, filling in the Chinese data gaps among global plant nutrient stoichiometry data. According to our statistics, 102 halophytic species were identified, accounting for nearly 13.50% of the total collected species. Thus, we also screened relevant data representing halophytes from paper published before 2005 for comparison with our newly collected dataset after 2010.

### 2.2 Data analysis

The arithmetic means of each species were calculated within each site. The frequency distribution of the leaf N and P concentrations, and N:P ratios were slightly skewed; therefore, we calculated the arithmetic means (**Figure S2**). We used one-way ANOVA followed by Tukey test to compare the leaf N and P concentrations and the N:P ratio among ecosystem types, life forms, and halophyte types. The linear regression analysis was performed to correlate the potential relationships between leaf N and P stoichiometry and geographical variables. Partial least squares path modeling (PLS-PM) was performed using the “plspm” package to identify the direct and indirect effects of geographical and climatic variables on leaf N and P stoichiometry. The Goodness-of-fit (GoF) was applied determining the overall fit degree of the model, and the GoF > 0.36 indicated high degree of the model fit (Wetzels et al., 2009). Standardized major axis regression (SMA) was used to examine the scaling relationship between leaf N and P concentrations among ecosystem types, life forms, and halophyte types. The linear mixed-effects model was used to disclose the associations between leaf N and P stoichiometry and ecosystem types (Bates et al., 2015). Hierarchical partitioning (HP) analysis was used to quantify the corresponding relative contributions of environmental variables to leaf N and P stoichiometry (Chevan and Sutherland, 1991). All statistical analyses were conducted using the R Software package, Version 4.1.2 (R foundation for Statistical Computing, Vienna, Austria). Figures and data visualization were performed using R language and GraphPad Prism version 9.0.0 for Windows (GraphPad Software, San Diego, CA).

## 3 Results

### 3.1 Geographical patterns of leaf N and P stoichiometry

The mean leaf N concentration of halophytes across China was 22.47 mg/g (n=288, standard deviation (SD)=9.43 mg/g), falling between those of the desert and grassland ecosystems across northern China, and those of wetland ecosystems across eastern coastal China and global coastal regions (**Table 1**; **Figure S2**). The mean leaf P concentration of halophytes across China was 1.78 mg/g (n=274, SD=0.77 mg/g), corresponding to those recorded for halophytes before 2005 across China and in 2013 in an arid saline environment in northwest China, while exceeded and fell short of the values recorded in the Chinese mainland and wetlands, respectively (**Table 1**; **Figure S2**). The average leaf N:P ratio was 13.79 (n=274, SD=5.40), close to those of the global mainland and wetlands but slightly below that obtained for halophytes before 2005 across China and in 2013 in an arid saline environment in northwest China (**Table 1**; **Figure S2**).

**Table 1:** Comparison of leaf N and P stoichiometry in halophytes across China with other plant species or flora across globe and China.

Ecosystem	Region	Time period (year)	Leaf N (mg/g)	Leaf P (mg/g)	Leaf N:P	Reference
Terrestrial ecosystem	Global	-2004	20.09	1.77	13.80	Reich and Oleksyn (2004)
Terrestrial ecosystem	China	-2005	20.20	1.45	16.30	Han et al. (2005)
Coastal wetland	Global	1980-2018	16.13	1.59	13.04	Hu et al. (2021)
Coastal and inland wetland	China	-2014	18.30	2.55	7.18	Hu et al. (2014)
Desert	Typical desert, North China	-2010	25.5	1.74	15.77	Li et al. (2010)
Grassland	Inner Mongolia Plateau, northern China	2002-2004	27.6	1.9	15.3	He et al. (2006); He et al. (2008)
Halophytes in desert	Arid Saline Environment, Northwest China	2013	28.12	1.85	15.37	Wang et al. (2015)
Halophytes in terrestrial ecosystem	China	-2005	24.35	1.74	16.29	Han et al. (2005)
Halophytes in coast, grassland and desert	China	2010-2021	22.47	1.78	13.79	This study

**Figure 2:**
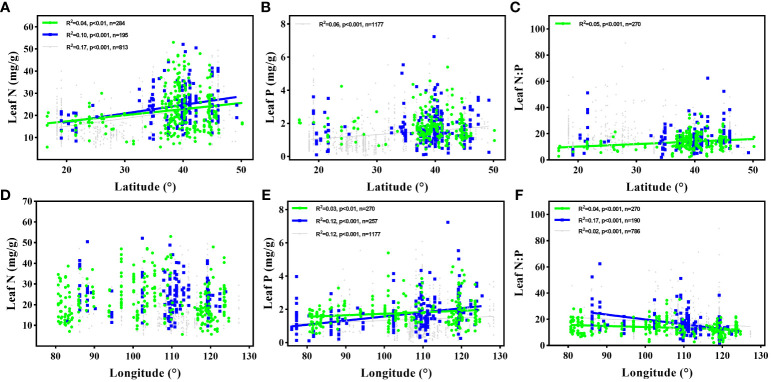
Variations in leaf N and P concentration, and N:P ratio of flora halophyte with latitude and longitude. The green points and lines represented the data from this study, and the blue and grey points and lines represented the data of halophytes and overall plants from Han et al. (2005), respectively. Each data point represents the species-specific average of all observations of N, P, or N:P within each sampling site. Linear regressions are shown for **(A)** latitude and leaf N in this study, halophytes in Han’s study, and overall plants in Han’s study; **(B)** latitude and leaf P of overall plants in Han’s study; **(C)** latitude and leaf N:P in this study; **(D)** longitude and leaf N; **(E)** longitude and leaf P in this study, halophytes in Han’s study, and overall plants in Han’s study; **(F)** longitude and leaf N:P in this study; **(D)** longitude and leaf N; **(E)** longitude and leaf P in this study, halophytes in Han’s study, and overall plants in Han’s study.

With an increase in LAT, the leaf N concentration and N:P ratio increased remarkably. The leaf P concentration and N:P ratio increased and decreased as the LON increased, respectively. No significant correlation was observed between the LAT and leaf P concentration or between the LON and leaf N concentration (**Figure 2**).

**Figure 3:**
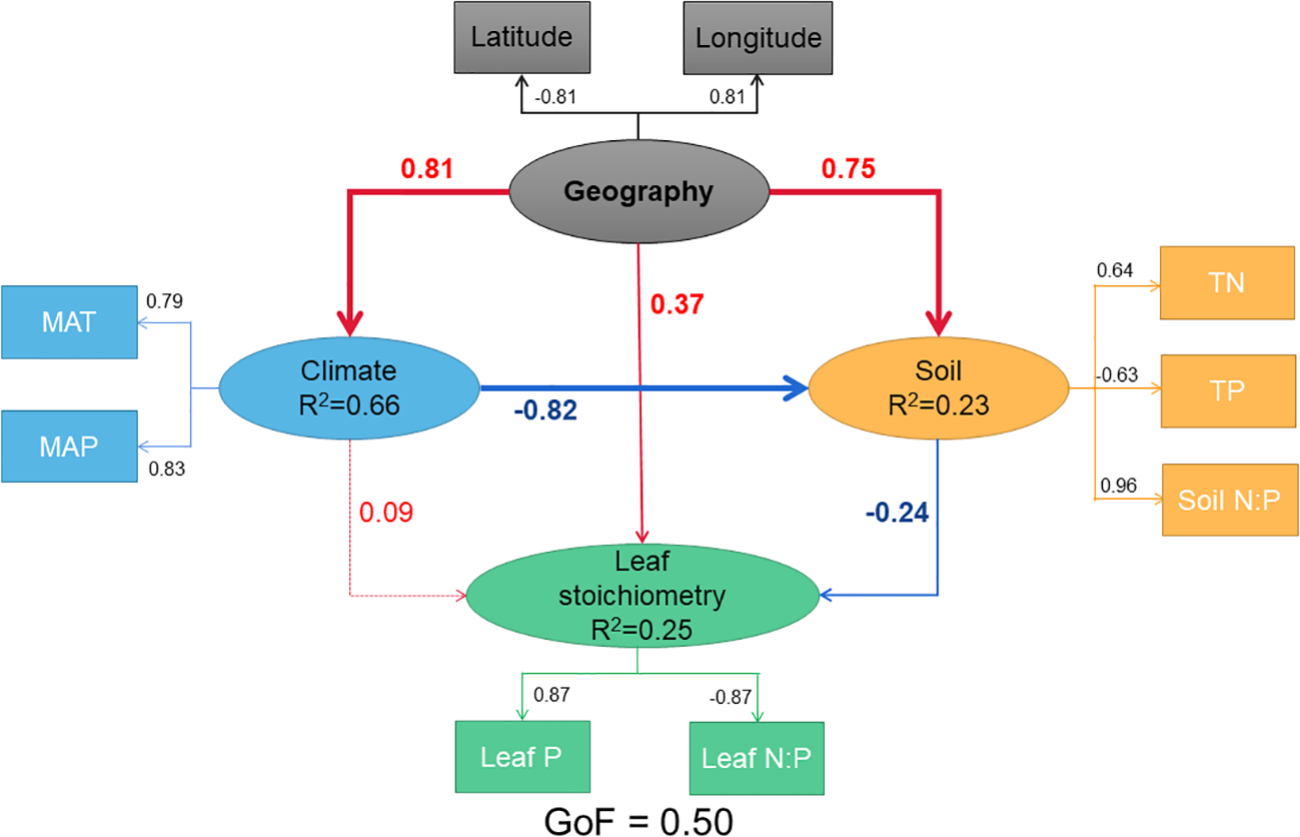
Predicted partial least squares path modeling (PLS-PM) for the direct and indirect associations between Geography and Leaf stoichiometry. Ellipse represents the structural model, and box represents corresponding measurement models. In the structural model, the lines indicated paths, and the values adjacent to the lines denote the magnitude of the path coefficients calculated by PLS regression. R^2^ values are shown for all endogenous latent variables in the ellipse. Values in the measurement model represent the loadings between a latent variable and its indicators. The figure is showing the final models after model diagnosis processes. Leaf N concentration and SOC (soil organic carbon) were removed because of the very low loadings (|loading| < 0.70), while we retained the TN and TP as their loadings closed to 0.70. The pseudo goodness-of-fit (GoF) of the model was 0.50, implying that the predicted model fit well. All paths are significant or marginally significant except the path from Climate to Leaf stoichiometry (p < 0.07).

According to **Figure 3**, the PLS-PM analysis results showed that geography (LAT and LON) had a significantly positive correlation (path coefficient=0.37, p=0.07 with a considerable trend toward significance) with leaf stoichiometry (leaf P concentration and N:P ratio), a significantly positive association (path coefficient=0.81, p<0.001) with climate (MAT and MAP), and a significantly positive association (path coefficient=0.75, p<0.001) with soil (TN, TP, and soil N:P ratio). The climatic variables had no association with leaf stoichiometry (path coefficient=0.09, p>0.05), while soil had a negative association with leaf stoichiometry (path coefficient=-0.24, p<0.05). The geographic, climatic and soil variables in the model explained 25.37% of the variance in the leaf stoichiometry. The percentage of the variance in climatic conditions explained by geography in the model was 66.38%. The percentage of the variance is soil conditions explained by geography and climate in the model was 23.46%. The direct associations (path coefficient=0.37) between geography and leaf stoichiometry were greater than the indirect associations (path coefficient=-0.02) of the paths with significant p-values, containing the latent variables of climate and soil, and the total association was 0.43.

### 3.2 Scaling relationships between leaf N and P concentrations

According to the results of SMA analysis, the leaf N and P concentrations of halophytes showed strong positive correlations in the pooled data among ecosystem types, growth forms, and halophyte types. The leaf P concentration increased with the leaf N concentration, exhibiting an SMA regression slope of 0.86 (p<0.001, **Table 2**). The regression slopes showed significant differences among halophyte types, and the slopes of euhalophytes, pseudohalophytes, and recretohalophytes were 0.40, 0.87, and 1.39, respectively (p<0.001, **Table 2**). Among ecosystem types, these slopes were 1.44, 1.01, and 0.73 for coastal, meadow, and desert ecosystems, respectively, reaching a marginal significance (p=0.07, **Table 2**). In addition, the slopes for herbs, shrubs, and trees exhibited no significant differences (p>0.05, **Table 2**).

**Table 2:** Standardized major axis regression slopes for log–log linear relationships between leaf N and P concentrations among all pooled data, ecosystem types, growth forms and halophyte types.

Group	n	R^2^	p	Slope	95% Cl
All	270	0.21	<0.001	1.17	0.93-1.48
Ecosystem type					
Coast	61	0.19	<0.001	0.69	0.35-1.19
Desert	160	0.21	<0.001	1.29	0.96-1.79
Meadow	49	0.63	<0.001	0.91	0.72-1.38
Growth form					
Herb	129	0.27	<0.001	1.12	0.84-1.51
Shrub	125	0.17	<0.001	1.28	0.86-2.00
Tree	16	0.39	<0.01	0.94	0.37-2.22
Halophyte type					
Euhalophyte	98	0.06	<0.05	2.50^c^	1.25-11.92
Pseudohalophyte	82	0.49	<0.001	1.15^b^	0.93-1.42
Recretohalophyte	90	0.16	<0.001	0.72^a^	0.39-1.20

### 3.3 Leaf N and P stoichiometry among different ecosystem types, plant growth forms, and halophyte types

**Figure 4:**
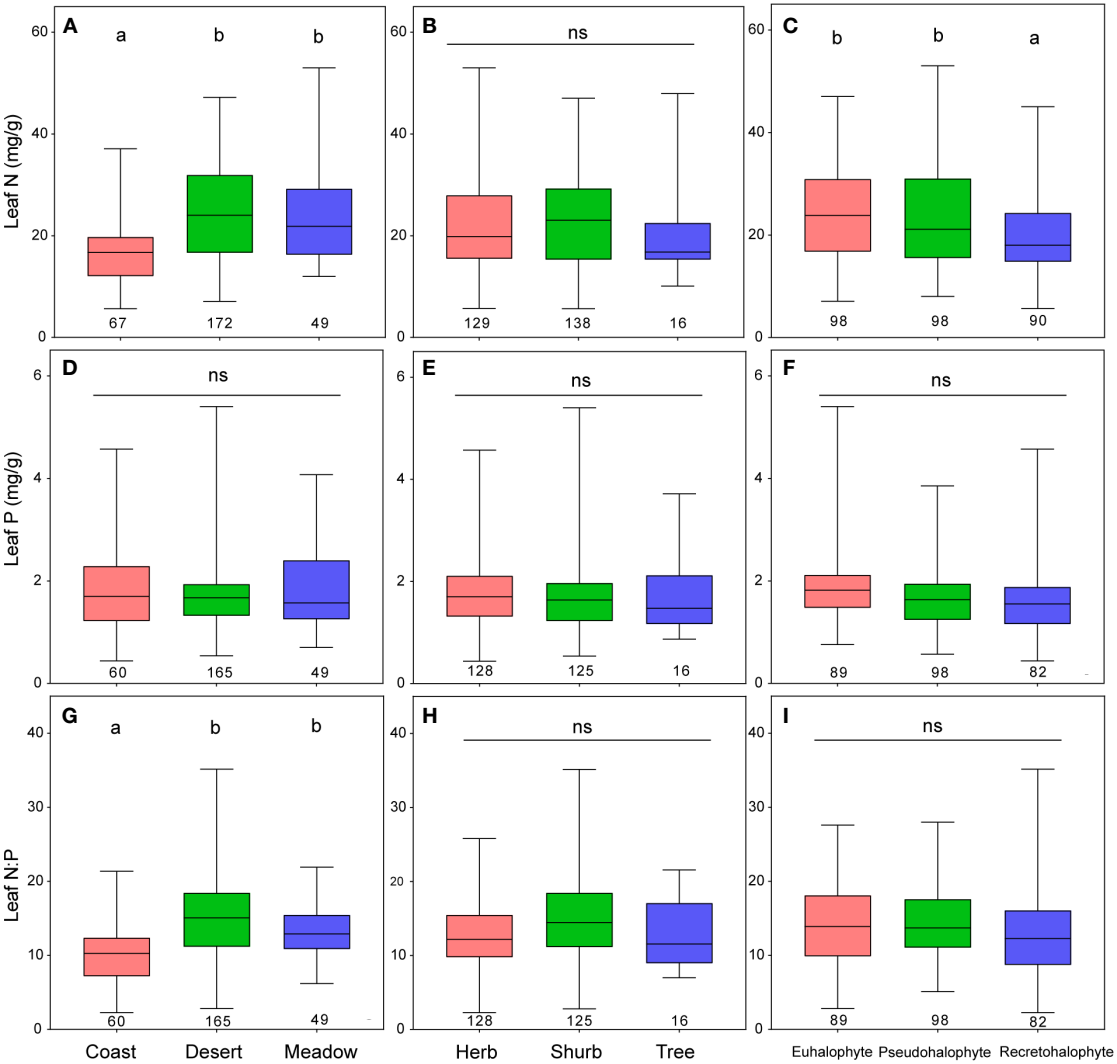
Leaf N and P stoichiometry among ecosystem types **(A, D, G)**, plant forms **(B, E, H)**, and halophyte types **(C, F, I)**. Different letters and ns mean significance (p<0.05) and no significance (p>0.05), respectively. The numbers under the boxplot are the sample size for each group.

The leaf N concentrations of desert and meadow halophytes were remarkably higher than those of coast halophytes across the pooled data, plant forms, and halophyte types, except for trees and pseudohalophytes (**Figures 4**, **S4**). The leaf P concentrations of halophytes remained relatively stable, except for recretohalophytes, across the pooled data, plant forms, and halophyte types. The leaf N:P ratio of desert halophytes was significantly higher than that of coastal halophytes among the plant forms and halophyte types (**Figures 4**, **S4**). After accounting for growth form and halophyte type, the impacts of the ecosystem type on the leaf N and P concentrations and the N:P ratio were significant, except for the impact on leaf P concentration considering the growth form (**Table 3**; **Figure S6**).

**Table 3:** Summary of linear mixed-effect models for the effects of ecosystem type on leaf N and P stoichiometry after accounting for growth form and halophyte type.

Variable		Fixed effects estimate	Marginal R^2^ without ecosystem type	Marginal R^2^ with ecosystem type	n	χ^2^	p
Leaf N	Growth form	1.27	0.01	0.12	284	19.92	**p<0.001**
	Halophyte type	1.30	0.03	0.12	284	15.15	**p<0.001**
	Growth form+ Halophyte type	1.28	0.03	0.12	284	14.47	**p<0.001**
Leaf P	Growth form	0.25	0.01	0.05	270	4.05	0.13
	Halophyte type	0.24	0.01	0.07	270	9.13	**0.01**
	Growth form+ Halophyte type	0.27	0.03	0.07	270	6.11	**0.05**
Leaf N:P	Growth form	1.03	0.03	0.15	270	19.13	**p<0.001**
	Halophyte type	1.06	0.01	0.15	270	24.18	**p<0.001**
	Growth form+ Halophyte type	1.01	0.05	0.16	270	16.81	**p<0.001**

Marginal R^2^ is the variance explained by the fixed-effect terms in the model, and n is the number of species–site combinations. Values of χ^2^ and p are from the likelihood ratio tests for comparing the full model including ecosystem type and listed variables and the reduced model without ecosystem type. Significant effects (p<0.05) are marked in bold.

## 4 Discussion

### 4.1 Geographical patterns of leaf N and P stoichiometry of halophytes

Understandings the geographical patterns of leaf N and P stoichiometry contribute to the development of nutrient flux models and the prediction of plant traits responses to global changes (Tian et al., 2019; Qin et al., 2022). In this study, the leaf N concentration exhibited a latitudinal pattern, mainly induced by temperature and precipitation variations occurring alongside LAT changes (**Figures 2**, **S2**; **Table S1**). This finding was consistent with previous observations of individual species or multispecies collections conducted at regional and global scales (Tong et al., 2021; Dynarski et al., 2023), and was in accord with the description of TPPH that plants required high N concentrations at low temperatures to compensate for the reduction of physiological efficiency (Reich and Oleksyn, 2004). In addition, the leaf P concentration exhibited a longitudinal pattern that may have been mainly induced by the variation in the soil TP content alongside LON changes (**Figures 2**, **S2**; **Table S1**). It is well known that close relationships generally exist between leaf P concentration and soil TP content, and this might be because the P required for plant growth and development is mainly obtained from the soil P pool in the natural state (Hedin, 2004).

We also observed that the leaf N:P ratio exhibited latitudinal and longitudinal patterns driven by the variations in temperature and precipitation by LAT, as well as the variation in soil P content by LON (**Figures 2**, **S3**; **Table S2**). Notably, the HP analysis suggested that the soil P content is likely responsible for the variation in the leaf N:P ratio when compared to MAT and MAP (**Figure S6**). These results confirmed the findings of Richardson et al. (2008) for multiple species in cool-temperate New Zealand rainforest.

Our results also displayed that the leaf N and P stoichiometry exhibited no latitudinal or longitudinal patterns in coastal halophytes. This finding was not in line with Hu et al. (2021) for salt marshes and mangroves across coastal wetlands at the global scale, which might be due to the difference in the study scale between the two studies. In addition, we observed that leaf N and P concentrations decreased with increasing LAT and LON for meadow halophytes. This finding was not in line with the overall results of the study whereas appeared to be consistent with the field observations of plant community in the HunLunbeier Grassland (Ding et al., 2012). Consequently, the overall patterns of leaf N and P stoichiometry patterns in halophytes were strongly controlled by large-scale geographical factors but simultaneously exhibited variations among diverse geographical locations.

### 4.2 Trade-off between N and P uptake in halophytes

N and P are the most limiting elements for plant growth in terrestrial ecosystems, and the allometric relationship between leaf N and P concentrations is considered a reflection of the pattern of nutrient allocation and plants’ resource-acquiring strategy (Broadbent et al., 2014; Tian et al., 2018; Yang et al., 2023). In this study, a positive coupling relationship has often been observed between N and P due to their joint roles in regulating biological growth, respiration, decomposition, and the biochemical cycles in ecosystems (Reich et al., 2010; Tian et al., 2018). In this study, the leaf N and P concentrations of halophytes exhibited significantly positive associations with each other across all pooled data, ecosystem types, plant forms, and halophyte types. Regarding the pooled data, the N and P scaling exponent was 1.16 (95% confidence interval: 0.93-1.48), indicating a relatively fast change in leaf N concentration compared to leaf P concentration. The N and P scaling exponent of halophytes was higher than the empirical values (i.e., 2/3 and 3/4), suggesting that halophytes might have special nutrient investment mechanisms compared to global plants due to their specific ecological niche (Niklas et al., 2005; Reich et al., 2010).

Furthermore, we explored the effects of abiotic and biotic factors on the N and P scaling exponents of halophytes, such as the ecosystem type, plant form, and halophyte type. The results showed that the N and P scaling exponents of halophytes could be ranked from large to small in desert, meadow, and coastal ecosystems, barely reaching marginally significance among ecosystem types; this finding indicated that the differences in temperature and precipitation among ecosystem types might alter the trade-off between N and P uptake when adapting to environmental changes (Lukac et al., 2010; Tian et al., 2018). No significant differences in the N and P scaling exponents of halophytes were observed among plant forms, which was inconsistent with the findings that the N and P scaling exponents of the trees were significantly higher than those of shrubs and herbs among global coastal wetland plants (Hu et al., 2021). Thus, further research is needed to explore whether the N and P scaling exponent is affected by the sample size or sample source/composition to some extent (Tian et al., 2018).

Previous studies have widely reported differences among the salt tolerance mechanisms of the three halophyte types from the perspective of their morphological features, osmotic balance regulation, and gene expression (Ibrahimova et al., 2021; Rahman et al., 2021; Meena et al., 2023). In the current study, we found that the N and P scaling exponents varied significantly among halophyte types, implying that the trade-off between N and P uptake exhibited large variations. Thus, the N and P scaling exponent could be applied as a functional property to reflect how plant nutrient availability responds to genetic differentiation in halophytes. In addition, these findings confirm that the application of ecological stoichiometry has great potential of systematic evolution and ecological adaptation (Elser et al., 2000; Jeyasingh and Weider, 2007).

### 4.3 Variation in leaf N and P stoichiometry of halophytes among different functional groups

The leaf nutrient concentrations are important indicators of plant growth status and habitat conditions. In this study, no notable differences were observed between leaf N and P concentrations in halophytes and those of global or national plants, which might be due to that halophytes maintain the nutrient balance to ensure normal life activities by different ways (Song et al., 2022; Zhao et al., 2022). The leaf N:P ratio has been used a threshold of the N or P limitation for plant growth (i.e. N limitation when N:P < 14, P limitation when N:P > 16) (Koerselman and Meuleman, 1996; Tessier and Raynal, 2003). It was observed that the mean leaf N:P ratio after 2010 was 13.39, remarkably lower than 16.29 obtained before 2005, revealing that the P limitation of plant growth was mitigated to some extent as time went on. This result might be due to the strengthen soil P replenishment by weathering of mineral rocks in recent years.

No significant difference was observed in leaf N and P stoichiometry among ecosystem types, plant forms, or halophyte types, except for the leaf N concentration among ecosystem types and halophyte types. This could be because the spatial distribution of halophytes was relatively narrow, leading to a low differentiation level of leaf nutrient trait. The leaf N concentrations of desert and meadow halophytes was significantly higher than those of coastal halophytes, which could be attributed to the fact that plants required more nutrients in the low-temperature areas (Reich and Oleksyn, 2004).

In this study, the leaf N concentrations of euhalophytes and pseudohalophytes were remarkably higher than those of recretohalophytes. This might be because of that differing from recretohalophytes which can alleviate salt injury in an autocrine manner, euhalophytes and pseudohalophytes require a great deal of protein to form the protoplasm or soluble material to maintain a high internal osmotic pressure. Overall, the leaf N concentration and N-P scaling exponent exhibited relatively high heterogeneity among recretohalophytes, euhalophytes, and pseudohalophytes, might providing credible ecological references for the division of halophyte types from the perspective of nutrient resource allocation, as well as shedding insights into plant adaptation strategies to special habitats.

Moreover, the leaf N and stoichiometry between phylogenetic development and photosynthetic pathway were also compared (**Figure S5**). It was observed that leaf N and P concentrations were both higher in dicotyledon plants than these in monocotyledon plants, which were also found in herbaceous and woody angiosperm species across 530 sites in China (An et al., 2021). This might be attributed to that dicotyledon plants could better absorb the nutrient from soils than monocotyledon plants. Generally, C3 plants have higher leaf N and P concentrations than C4 plants (Han et al., 2005; Zhang et al., 2010), however, this was not confirmed for halophytes in our study.

### 4.4 Meaning and limitation

In this study, we explored the spatial distribution characteristics of leaf nutrients in halophytes using the ecological stoichiometry theory. Our results displayed that the leaf N and P concentrations in halophytes exhibited significantly latitudinal and longitudinal patterns, respectively, which had been widely confirmed in previous studies, supporting the views that the differences between halophytes and non-halophytes in response to salinity were expressed quantitatively (or by degree) rather than qualitatively.

It was observed that the leaf N:P of halophytes was 13.79, slightly lower than the N-limitation threshold value, which suggested that the plant growth of halophytes was likely not limited by soil nutrient deficiencies. The leaf N concentration and N-P scaling exponent exhibited differences among halophyte types, which would be a guideline for the taxonomic classification of this special plant group. Overall, our work would help draw attention to the spatial and temporal distribution patterns and allocation strategies of nutrients in halophytes at the national scale, as well as the other special plant resources, and further providing beneficial scientific support for the nutrient management in the saline-alkali grain cultivation.

However, there are several limitations in this study as well. In details, it is well known that halophytes have developed specialized molecular mechanisms and cellular structures to tolerate high salt stress, and these adaptations might induce the changes in plant nutrient allocation strategies. In this study, we just observed that leaf N concentration increased with soil pH, which was in line with previous studies (**Figure S1**) (Zhang et al., 2019; Gong et al., 2020). We also found that soil salinity had no effects on leaf N and P stoichiometry (**Figure S1**). This finding was consistent with the results of prior studies for halophytes in an arid saline environment, Northwest China, however further research is needed to explore the physiological mechanisms of nutrient stability within leaves under high salinity levels (Wang et al., 2015). Further, some other key indicators such as soil Na+ content and EC value were not included in the data analysis due to insufficient data. Therefore, field sampling should be conducted to obtain evidence regarding the causal relationships between salt stress and nutrient characteristics in subsequent studies.

## 5 Conclusions

As a special germplasm resource with an extremely important strategic significance, halophytes should be fully characterized from different perspectives. Here, we explored the spatial distribution patterns and allocation strategies in the leaf N and P stoichiometry of halophytes at the national scale using the data integration analysis. The leaf N and P stoichiometry of halophytes exhibited clear biogeographical patterns, and mainly driven by temperature and soil P content. The leaf N concentration and N-P scaling exponent could serve as a reference for the type division of halophytes. In summary, our results have provided new insights into the plant ecological adaptation especially with special habitats and the basic information on the nutrient management for the halophytes cultivation.

## Data availability statement

The original contributions presented in the study are included in the article/**Supplementary Material**. Further inquiries can be directed to the corresponding authors.

## Author contributions

RT: Methodology, Conceptualization, Data curation, Software, Writing – original draft. CM: Data curation, Writing – review & editing. CL: Data curation, Writing – review & editing. WY: Data curation, Software, Writing – original draft. NZ: Data curation, Software, Writing – original draft. GW: Methodology, Writing – review & editing. TW: Methodology, Writing – review & editing, Project administration, Resources, Supervision.
