# Supplementary material for: Leaf nitrogen and phosphorus stoichiometry of the halophytes across China
Source: Front Plant Sci. 2023 Oct 4;14:1276699. doi: 10.3389/fpls.2023.1276699 (PMC10582939; doi:10.3389/fpls.2023.1276699)
Supplement: Supplementary file 1 [file DataSheet_1.docx]

Text S1

1 Abdurahman, A. (2015). Ecological stoichiometry characteristics of two species of halophytes and their habitat soil under different moisture-fertilization gradients (Master), Xinjiang University, Xinjiang. (In Chinese)

2 Bao, H., Qing, H., Wang, L. X., Duan, Y. X., Liu, W. D., Zhuo, Y., Liu, H. M. (2014). A study on ecological stoichiometry of plants on lakeside zone of Wuliangsuhai Lake. Journal of Inner Mongolia University (Natural Science Edition), **45**(4), 404-409. (In Chinese)

3 Cao, C., Jian, S. G., Ren, H., Wang, J., Liu, N., Wang, J. X., Wu, S. H. (2017). The ecophysiological characteristics of *Pemphis acidula*, a tropical beach plant. Ecology and Environment Sciences, **26**(12), 2064-2070. (In Chinese)

4 Cao, S. K., Feng, Q., Si, J. H., Zhang, X. F., Liu, W., Chang, Z. Q. (2011). Status of foliar nutrients in *Populus euphratica* desert riparian forest in Northwestern China. Journal of Desert Research, 31(**5)**. 1131-1140. (In Chinese)

5 Cao, S. K., Feng, Q., Su, Y. H., Chang, Z. Q., Xi, H. Y. (2011). Research on the water use efficiency and foliar nutrient status of *Populus euphratica* and *Tamarix ramosissima* in the extreme arid region of China. Environmental Earth Sciences, **62**(8), 1597-1607. (In Chinese)

6 Ding, F., Lian, P. Y., Zeng, D. H. (2011). Characteristics of plant leaf nitrogen and phosphorus stoichiometry in relation to soil nitrogen and phosphorus concentrations in Songnen Plain meadow. Chinese Journal of Ecology, **30**(1), 77-81. (In Chinese)

7 Fan, Q. C., Xie, W. X., Wang, Z. Q., Li, P. (2019). Seasonal variations in C, N and P stoichiometry of *Spartina alterniflora*. Environmental Science and Technology, **42**(4), 12-19. (In Chinese)

8 Fan, Y., Pan, Y. L., Chen, Z. W., Lin, H., Xu, R., Wu, C. Z., Hong, T. (2019). C: N: P stoichiometry in roots, stems, and leaves of four mangrove species. Chinese Journal of Ecology, **38**(4), 1041-1048. (In Chinese)

9 Fu, X. Y. (2012). Studies on leaf traits of alashan desert plants (Master), Inner Mongolia University, Inner Mongolia. (In Chinese)

10 Gong, X. W., Lv, G. H., Ma, Y., Zhang, X. N., He, X. M., Guo, Z. J. (2017). Ecological stoichiometry characteristics in the soil under crown and leaves of two desert halophytes with soil salinity gradients in Ebinur Lake Basin. Scientia Silvae Sinicae, **53**(4), 28-36. (In Chinese)

11 He, M. Z., Zhang, K., Tan, H. J., Hu, R., Su, J. Q., Wang, J., Huang, L., Zhang, Y.F., Li, X. R. (2015). Nutrient levels within leaves, stems, and roots of the xeric species Reaumuria soongorica in relation to geographical, climatic, and soil conditions. Ecology and evolution, **5**(7), 1494-1503.

12 Huang, G., Su, Y. G., Mu, X. H., Li, Y. (2018). Foliar nutrient resorption responses of three life-form plants to water and nitrogen additions in a temperate desert. Plant and Soil, **424**(1), 479-489.

13 Jiang, L. L., Zeng, C. S., Shao, J. J., Zhou, X. H. (2017). Plant nutrient dynamics and stoichiometric homeostasis of invasive species *Spartina alterniflora* and native *Cyperus malaccensis* var. brevifolius in the Minjiang River estuarine wetlands. Chinese Journal of Plant Ecology, **41**(4), 450-460. (In Chinese)

14 Jin, Z. Z., Xu, X. W., Li, S. Y., Zhong, X. B., Yan, J., Wang, G. F., Yang, A. Y. (2013). Stoichiometric characteristics of fresh leaves in several typical desert plants at the hinterland of Taklimakan desert. Environment, Energy and Sustainable Development, **4**, 93.

15 Li, J. (2017). Plant population dynamics and ecological stoichiometry of Mangrove in Beilun Estuary in Guangxi. (Master), Guangxi Normal University, Guangxi. (In Chinese)

16 Li, C. J., Lei, J. Q., Xu, X. W., Tang, Q. L., Gao, P., Wang, Y. D. (2013). The stoichiometric characteristics of C, N, P for artificial plants and soil in the hinterland of Taklimakan Desert. Acta Ecologica Sinica, **33**(18), 5760-5767. (In Chinese)

17 Li, S. J., Gou, W., Wang, H., Wu, G. Q., Su, P. X. (2019). Characteristics of C, N, P, and their response to soil water and salt in leaves of Lycium ruthenicum in the lower reaches of the Heihe River. Acta Ecologica Sinica, **39**(19), 7189-7196. (In Chinese)

18 Li, H. L., Gong, L., Hong, Y. (2016). Seasonal variations in C, N, and P stoichiometry of roots, stems, and leaves of Phragmites australis in the Keriya Oasis, Xinjiang, China. Acta Ecologica Sinica, **36**(20), 6547-6555. (In Chinese)

19 Liu, J. H. (2021). Ecological stoichiometric characteristics of typical halophytes under different salinity gradients in the Yellow River Delta. (Master), Shandong Agricultural University, Shandong. (In Chinese)

20 Liu, W. L. (2014). Carbon, nitrogen and phosphorus content and ecological stoichiometric characteristics of the Jiaozhou Bay Wetland Ecosystem. (Master), Qingdao University, Shandong. (In Chinese)

21 Liu, Q., Sun, J. K., Tian, J. Y., Zhang, M. (2010). Nutrients absorption and cumulation in typical edificatos of seashell islands in Yellow River Delta. Research of Soil and Water Conservation, **17**(3), 153-156, 161. (In Chinese)

22 Liu, P., Ma, H., Zhi, Y. B., Cui, Y., Sun, A. A., Gou, Y. N., Li, Q., Gao, T.Y., Zhang, H.L., Liu, H. Y. (2018). Ecological stoichiometric differences of nine typical eremophyte species. Arid Zone Research, **35**(1), 207-216. (In Chinese)

23 Liu, F. D., Zheng, Y., Liu, Y. H., Dong, Y. F., Li, D. S., Wang, M. H. (2019). Stoichiometric nutrient balance of Suaeda salsa wetlands in different supratidal habitats of Tianjin, China. Hydrobiologia, **827**(1), 3-19.

24 Liu, X. H., Gong, Y. Q., Chen, W. F., Huang, B. H., Zhu, R. S. (2018). C, N and P stoichiometry of typical plants and soils in the Yellow River Delta Natural Reserve. Chinese Journal of Eco-Agriculture, **26**(11), 1720-1729. (In Chinese)

25 Luo, Y., Gong, L., Zhu, M. L., An, S. Q. (2017). Stoichiometry characteristics of leaves and soil of four shrubs in the upper reaches of the Tarim River Desert. Acta Ecologica Sinica, **37**(24), 8326-8335. (In Chinese)

26 Niu, D. C., Li, Q., Jiang, S. G., Chang, P. J., Fu, H. (2013). Seasonal variations of leaf C:N:P stoichiometry of six shrubs in desert of China’s Alxa Plateau. Chinese Journal of Plant Ecology, **37**(4), 317-325. (In Chinese)

27 OuYang, P. Y., Liu, N., Zhang, W. W., Wang, J., Jian, S. G. (2011). Biological and ecophysiologicai characteristics of a beach plant Ipomoea pescaprae. Journal of Hunan University of Science & Technology（Natural Science Edition), **26**(4), 117-121. (In Chinese)

28 Rong, Q. Q., Liu, J. T., Cai, Y. P., Lu, Z. H., Zhao, Z. Z., Yue, W. C., Xia, J. B. (2015). Leaf carbon, nitrogen and phosphorus stoichiometry of *Tamarix chinensis* Lour. in the Laizhou Bay coastal wetland, China. Ecological Engineering, **76**, 57-65. (In Chinese)

29 Shi, J. H., Wang, X. Y., Liu, M. X., Ma, X. X. (2017). Stoichiometric Characteristics of leaves of *Populus euphratica* with different stand ages and soil. Arid Zone Research, **34**(4), 815-822. (In Chinese)

30 Song, Y. T., Zhou, D. W., Li, Q., Wang, P., Huang, Y. X. (2012). Leaf nitrogen and phosphorus stoichiometry in 80 herbaceous plant species of Songnen grassland in Northeast China. Chinese Journal of Plant Ecology, **36**(3), 222-230. (In Chinese)

31 Song, G. M., Liu, N., Jian, S. G., Liu, H., Zhang, W., Han, T. T., Wang, J., Ren, H. (2018). Physiological and biological characteristics of *Terminalia catappa*. Journal of Tropical and Subtropical Botany, **26**(1), 40-46. (In Chinese)

32 Sun, L., Gong, L., Zhu, M. L., Xie, L. N., Li, H. L., & Lou, Y. (2017). Leaf stoichiometric characteristics of typical desert plants and their relationships to soil environmental factors in the northern margin of the Tarin Basin. Chinese Journal of Ecology, **36**(5), 1208-1214. (In Chinese)

33 Sun, J., Xu, H. M., Zhao, W. Y., Zan, Q. J., Chen, L. E., Liao, W. B. (2016). Ecological and biological characteristics of Myoporum bontioides, a semi-mangrove plant in China. Journal of Tropical Oceanography, **35**(6), 58-67. (In Chinese)

34 Tan, H. X., Jin, Z. G., Sun, F. Q., Peng, H. L., Yang, Z., & Geng, S. G. (2018). Stoichiometry characteristics of typical halophytes in Luanhe Estuary Wetland. Bulletin of Botanical Research, **38**(6), 956-960. (In Chinese)

35 Tao, W. (2016). Study on growth ecological stoichiometry relation of *Suaeda heteropterain* Liaohe River Estuary wetland. (Master), Dalian Ocean University, Liaoning. (In Chinese)

36 Wang, L. L., Zhao, G. X., Li, M., Zhang, M. T., Zhang, L. F., Zhang, X. F., An, L.Z., Xu, S. J. (2015). C: N: P stoichiometry and leaf traits of halophytes in an arid saline environment, northwest China. PLoS One, **10**(3), e0119935.

37 Wang, L. P., Zhang, J., Geng, Y. J., Ge, S. Q., Sun, W., Fan, S. P. (2018). Ecological stoichiometry characteristics of carbon, nitrogen and phosphorus of *Phragmites australis* leaves in Wowachi Wetland and their influence factors. Wetland Science, **16**(3), 417-423. (In Chinese)

38 Wang, W. Q., Xu, L. L., Zeng, C. S., Tong, C., Zhang, L. H. (2011). Carbon, nitrogen and phosphorus ecological stoichiometric ratios among live plant-litter-soil systems in estuarine wetland. Acta Ecologica Sinica, **31**(23), 7119-7124. (In Chinese)

39 Wang, H. Y., Wang, Z. W., Ding, R., Hou, S. L., Yang, G. J., Lü, X. T., Han, X. G. (2018). The impacts of nitrogen deposition on community N: P stoichiometry do not depend on phosphorus availability in a temperate meadow steppe. Environmental pollution, **242**, 82-89.

40 Wei, S. D., Liu, X. W., Zhang, L. H., Chen, H., Zhang, H., Zhou, H. C., Lin, Y. M. (2015). Seasonal changes of nutrient levels and nutrient resorption in *Avicennia marina* leaves in Yingluo Bay, China. Southern Forests: a Journal of Forest Science, **77**(3), 237-242.

41 Wu, T. G., Wu, M., Liu, L., Xiao, J. H. (2010). Seasonal variations of leaf nitrogen and phosphorus stoichiometry of three herbaceous species in Hangzhou Bay coastal wetlands, China. Chinese Journal of Plant Ecology, **34**(1), 23-28. (In Chinese)

42 Xiao, Y., Tao, Y., Zhang, Y. M. (2014). Biomass allocation and leaf stoichiometric characteristics in four desert herbaceous plants during different growth periods in the Gurbantünggüt Desert, China. Chinese Journal of Plant Ecology(9), 929-940. (In Chinese)

43 Xie, L. P., Wang, B. D., Xin, M., Wang, M., He, X. P., Wei, Q. S., Shi, X.Y., Sun, X. (2019). Characteristics of vegetation carbon, nitrogen, and C/N ratio in a *Tamarix chinensis* coastal wetland of China. Clean–Soil, Air, Water, **47**(9), 1800452.

44 Xu, B. B., Liu, N., Ren, H., Wang, X. H., Liu, N., Jian, S. G. (2018). Stress resistance biological characteristics of Scaevola sericea in Paracel Islands. Guihaia, **38**(10), 1277-1285. (In Chinese)

45 Yang, Y., Liu, B. R., An, S. S. (2018). Ecological stoichiometry in leaves, roots, litters and soil among different plant communities in a desertified region of Northern China. Catena, **166**, 328-338.

46 Yang, X., Li, Q., Wang, S. M., Hou, B. L., Zhang, J. Q., Wang, G. (2011). Stoichiometric analysis of leaves of two types of *Nitraria* and the sited *Nebkhas* soil. Journal of Desert Research, **31**(5), 1156-1161. (In Chinese)

47 Yong, Y. H., Zhang, X., Wang, S. M., Wu, L. (2016). Salt accumulation in vegetative organs and ecological stoichiometry characteristics in typical halophytes in Xinjiang, China. Chinese Journal of Plant Ecology, **40**(12), 1267-1275. (In Chinese)

48 Zeng, C. S., Zhang, L. H., Tong, C. (2009). Seasonal variation of nitrogen and phosphorus concentration and accumulation of Cyperus malaccensis in Minjiang River estuary. Chinese Journal of Ecology, **28**(5), 788-794. (In Chinese)

49 Zhang, B., Gao, X. P., Li, L., Lu, Y., Shareef, M., Huang, C. B., Liu, G.J., Gui, D.W., Zeng, F. J. (2018). Groundwater depth affects phosphorus but not carbon and nitrogen concentrations of a desert phreatophyte in Northwest China. Frontiers in plant Science, **9**, 338.

50 Zhang, L. F., Wang, L. L., He, W. L., Zhang, X. F., An, L. Z., Xu, S. J. (2018). Patterns of leaf N: P stoichiometry along climatic gradients in sandy region, north of China. Journal of Plant Ecology, **11**(2), 218-225.

51 Zhang, W. W., Liu, N., Wang, J., Ren, H., Zhang, L. M., Jian, S. G. (2012). Ecological and biological characteristics of *Hibiscus tiliaceus*, a mangrove associate in China. Guihaia, **32**(2), 198-202. (In Chinese)

52 Zhang, S. Q., Xu, Q., Yao, H. R., Yang, Q., Liu, W. J., Wang, M. (2020). Carbon, nitrogen and phosphorus contents and their ecological stoichiometry characteristics in leaves of C*asuarina equisetifolia* and *Ipomoea pes-caprae* in the coastal zone of Hainan Island. Bulletin of Botanical Research, **40**(2), 224-232. (In Chinese)

53 Zhang, X. L., Guan, T. Y., Zhou, J. H., Cai, W. T., Gao, N. N., Du, H., Jiang, L.H., Lai, L.M., Zheng, Y. R. (2018). Community characteristics and leaf stoichiometric traits of desert ecosystems regulated by precipitation and soil in an arid area of China. International journal of environmental research and public health, **15**(1), 109.

54 Zhang, S. K., Huang, Y., Jian, S. G., Liu, N. (2019). Stress resistance characteristics of *Calophyllum inophyllum*, a tropical beach plant. Journal of Tropical and Subtropical Botany, **27**(4), 391-398. (In Chinese)

55 Zhang, J., Liu, Y. H., Sheng, J. D., Chai, Q., Li, R. X., Zhao, D. (2018). Carbon and nitrogen traits of typical shrubs in grassland of northern Xinjiang, China. Chinese Journal of Plant Ecology, **42**(3), 307-316. (In Chinese)

56 Zhang, K., Su, Y. Z., Liu, T. N., Wang, T. (2016). Leaf C: N: P stoichiometrical and morphological traits of *Haloxylon ammodendron* over plantation age sequences in an oasis-desert ecotone in North China. Ecological research, **31**(3), 449-457. (In Chinese)

57 Zhang, X. L., Zhou, J. H., Lai, L. M., Jiang, L. H., Zheng, Y. R., Shi, L. J. (2019). Leaf traits and ecological stoichiometry of dominant desert species across oasis-Gobi desert ecotone in the lower reaches of Heihe River, China. Chinese Journal of Applied & Environmental Biology, **25**(6), 1270-1276. (In Chinese)

58 Zhao, S. L., Zeng, F. J., Zhang, B., Liu, B., Gao, H. H. (2017). Effects of different disturbance treatments on the stoichiometric characteristics of stems, leaves and assimilative branches of *Alhagi sparsifolia* Shap. Arid Zone Research, **34**(4), 837-846. (In Chinese)

59 Zhao, M. X., Li, D. Z., Pan, Y., Lü, Y. Y., Gao, J. J., Cheng, L. L. (2012). Ecological stoichiometrical analysis on the strategies of utilization of nitrogen and phosphorus in *Phragmites australis* and *Spartina alterni* flora in Chongming Dongtan wetland. Guihaia, **32**(6), 715-722. (In Chinese)

60 Zhou, L. L., Li, S. B., Wang, W. P., X.Y., Y., Chen, Z., Pan, H. (2020). Leaf C, N, P stoichiometry and nutrient resorption characteristics among four mangrove tree species in the Zhangjiangkou wetland, Fujian Province. Chinese Journal of Applied and Environmental Biology, **26**(3), 674-680. (In Chinese)

61 Zhou, X. B., Tao, Y., & Zhang, Y. M. (2018). The C, N and P stoichiometry of dominant species in different land use types in a desert-oasis ecotone of the Southern Taklimakan Desert. Acta Prataculturae Sinica, **27**(5), 15-26. (In Chinese)

62 Zhu, Q. L. (2017). Carbon, nitrogen and phosphorus ecological stoichiometry characteristics of herbs in the coastal wetland of Fujian, Zhejiang and Shanghai. (Master), Fujian Normal University, Fujian. (In Chinese)

63 Zhu, J. T., Li, X. Y., Zhang, X. M., Zeng, F. J., & Yang, S. G. (2010). Leaf functional traits of C*eratoides Latens* in northern slope of Kunlun Mountain and its regional difference with the Altitude. Journal of Desert Research, **30**(6), 1325-1330. (In Chinese)
