# Supplementary material for: Leaf nitrogen and phosphorus stoichiometry of the halophytes across China
Source: Front Plant Sci. 2023 Oct 4;14:1276699. doi: 10.3389/fpls.2023.1276699 (PMC10582939; doi:10.3389/fpls.2023.1276699)
Supplement: Supplementary file 2 [file DataSheet_2.docx]

**Supplementary**

**Text S1** A list of 63 papers from which the data were extracted for this integration analysis.

**Table S2** Relationships between geographical, climatic, and soil variances.

**Table S3** Relationships between latitude and leaf N and P concentrations, and N:P ratio of halophytes in the three ecosystem types.

**Table S4** Relationships between longitude and leaf N and P concentrations, and N:P ratio of halophytes in the three ecosystem types.

**Table S5** Summary of linear mixed-effect models for the effects of latitude on leaf N and P stoichiometry of halophytes after accounting for ecosystem type, growth form, and halophyte type.

**Table S6** Summary of linear mixed-effect models for the effects of longitude on leaf N and P stoichiometry of halophytes after accounting for ecosystem type, growth form, and halophyte type.

**Figure S1** Histograms showing the distribution of leaf nitrogen (mg/g) (a); phosphorus (mg/g) (b); N:P ratio (c). The curves indicate fitted normal curves.

**Figure S2** Scatter plots showing the relationships between leaf N and P stoichiometry, and climatic and soil variables.

**Figure S3** Leaf N and P stoichiometry of halophytes among ecosystem types for each plant form and halophyte type. Different letters and ns mean significance (p<0.05) and no significance (p>0.05), respectively. The numbers under the boxplot are the sample size for each group.

**Figure S4** Leaf N and P stoichiometry among phylogeny (dicotyledon and monocotyledon) and photosynthetic pathways (C3 and C4 herbs). Different letters and ns mean significance (p<0.05) and no significance (p>0.05), respectively. The numbers under the boxplot are the sample size for each group.

**Figure S5** Percentage of the total variance in leaf N and P stoichiometry of halophytes by climatic and soil variances.

**Table S2**

|  | Longitude | Latitude | MAT | MAP | SOC | TN | TP |
| --- | --- | --- | --- | --- | --- | --- | --- |
| MAT | 0.01 | **-0.91** |  |  |  |  |  |
| MAP | **0.57** | **-0.77** | **0.69** |  |  |  |  |
| SOC | 0.10 | -0.01 | -0.10 | -0.03 |  |  |  |
| TN | **0.48** | -0.04 | -0.18 | **0.29** | **0.23** |  |  |
| TP | **0.20** | **-0.35** | **0.39** | **0.28** | -0.08 | -0.01 |  |
| Soil N:P | **0.27** | 0.03 | **-0.24** | 0.14 | **0.55** | **0.68** | **-0.45** |

**Table S3**

|  | Coast | | | Desert | | | Meadow | | |
| --- | --- | --- | --- | --- | --- | --- | --- | --- | --- |
|  | Estimate | *R^2^* | p | Estimate | *R^2^* | p | Estimate | *R^2^* | p |
| Leaf N | 0.05 | 0.00 | 0.63 | -0.55 | 0.02 | 0.06 | -2.14 | 0.29 | **0.00** |
| Leaf P | 0.01 | 0.02 | 0.35 | -0.01 | 0.01 | 0.70 | -0.15 | 0.20 | **0.00** |
| Leaf N:P | 0.00 | 0.00 | 0.95 | -0.03 | 0.00 | 0.89 | -0.09 | 0.01 | 0.62 |

Note: Significant relationships are marked in bold.

**Table S4**

| Variable | Coast | | | Desert | | | Meadow | | |
| --- | --- | --- | --- | --- | --- | --- | --- | --- | --- |
|  | *Estimate* | *R*^2^ | *p* | *Estimate* | *R*^2^ | *p* | *Estimate* | *R*^2^ | *p* |
| Leaf N | 0.04 | 0.00 | 0.87 | 0.33 | 0.11 | **0.00** | -0.71 | 0.14 | **0.01** |
| Leaf P | 0.01 | 0.00 | 0.77 | 0.02 | 0.06 | **0.00** | -0.04 | 0.08 | **0.05** |
| Leaf N:P | 0.04 | 0.00 | 0.78 | 0.08 | 0.02 | 0.09 | -0.06 | 0.01 | 0.53 |

Note: Significant relationships are marked in bold.

**Table S5**

| Variable | | Fixed effects estimate | Marginal R^2^ with latitude | Marginal R^2^ without latitude | n | χ^2^ | p |
| --- | --- | --- | --- | --- | --- | --- | --- |
| Leaf N | Ecosystem type | 1.48 | 0.12 | 0.11 | 284 | 0.88 | 0.35 |
|  | Growth form | 1.27 | 0.03 | 0.01 | 284 | 5.05 | **0.03** |
|  | Halophyte type | 0.90 | 0.05 | 0.03 | 284 | 2.40 | 0.12 |
|  | Ecosystem type + Growth form + Halophyte type | 1.54 | 0.13 | 0.13 | 284 | 1.11 | 0.29 |
| Leaf P | Ecosystem type | 0.24 | 0.05 | 0.04 | 270 | 0.00 | 0.998 |
|  | Growth form | 0.40 | 0.01 | 0.01 | 270 | 0.33 | 0.567 |
|  | Halophyte type | 0.53 | 0.02 | 0.01 | 270 | 0.996 | 0.318 |
|  | Ecosystem type + Growth form + Halophyte type | 0.29 | 0.08 | 0.07 | 270 | 0.19 | 0.661 |
| Leaf N:P | Ecosystem type | 1.29 | 0.14 | 0.14 | 270 | 0.94 | 0.331 |
|  | Growth form | 0.35 | 0.06 | 0.03 | 270 | 4.98 | **<0.05** |
|  | Halophyte type | 0.39 | 0.05 | 0.01 | 270 | 4.63 | **<0.05** |
|  | Ecosystem type + Growth form + Halophyte type | 0.91 | 0.16 | 0.16 | 270 | 0.57 | 0.449 |

**Table S6**

| Variable | | Fixed effects estimate | Marginal R^2^ with longitude | Marginal R^2^ without longitude | n | χ^2^ | p |
| --- | --- | --- | --- | --- | --- | --- | --- |
| Leaf N | Ecosystem type | -0.10 | 0.13 | 0.11 | 284 | 2.38 | 0.12 |
|  | Growth form | 1.69 | 0.01 | 0.01 | 284 | 0.65 | 0.42 |
|  | Halophyte type | 1.30 | 0.03 | 0.03 | 284 | 0.62 | 0.43 |
|  | Ecosystem type + Growth form + Halophyte type | 0.09 | 0.14 | 0.13 | 284 | 1.68 | 0.20 |
| Leaf P | Ecosystem type | -0.39 | 0.05 | 0.05 | 270 | 0.47 | 0.49 |
|  | Growth form | -0.80 | 0.04 | 0.01 | 270 | 3.66 | 0.06 |
|  | Halophyte type | -1.07 | 0.05 | 0.01 | 270 | 6.52 | **0.01** |
|  | Ecosystem type + Growth form + Halophyte type | -0.00 | 0.07 | 0.07 | 270 | 0.11 | 0.74 |
| Leaf N:P | Ecosystem type | 0.42 | 0.14 | 0.14 | 270 | 0.31 | 0.58 |
|  | Growth form | 2.37 | 0.08 | 0.03 | 270 | 45.37 | **<0.001** |
|  | Halophyte type | 2.75 | 0.07 | 0.01 | 270 | 9.05 | **<0.01** |
|  | Ecosystem type + Growth form + Halophyte type | 0.23 | 0.16 | 0.16 | 270 | 0.52 | 0.47 |

**
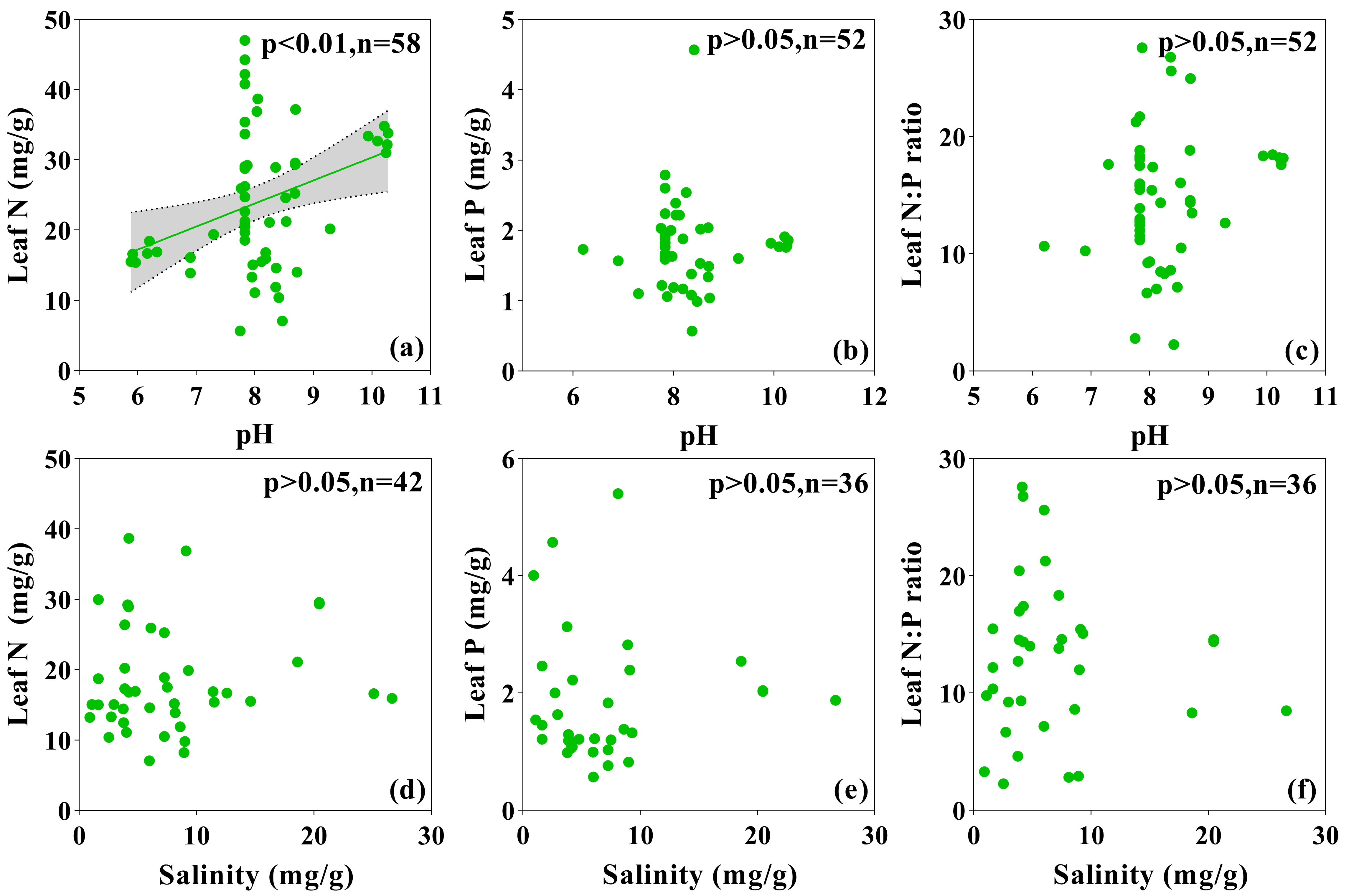
**

**Figure S1**

**
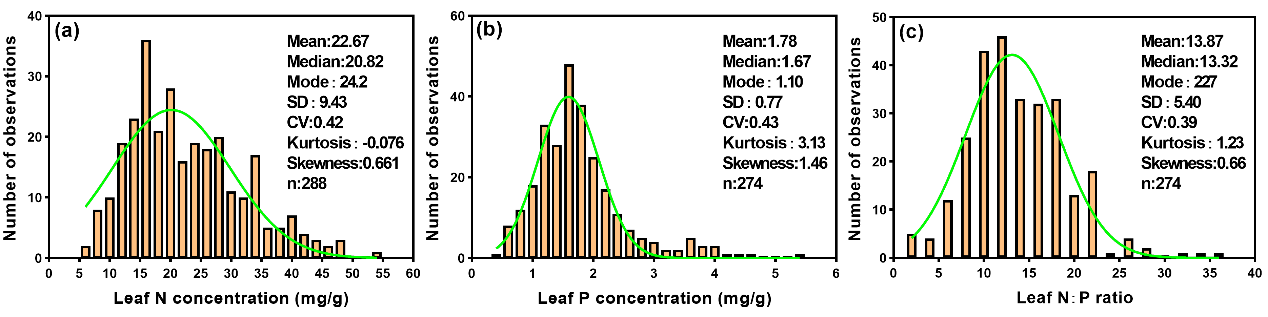
**

**Figure S2**


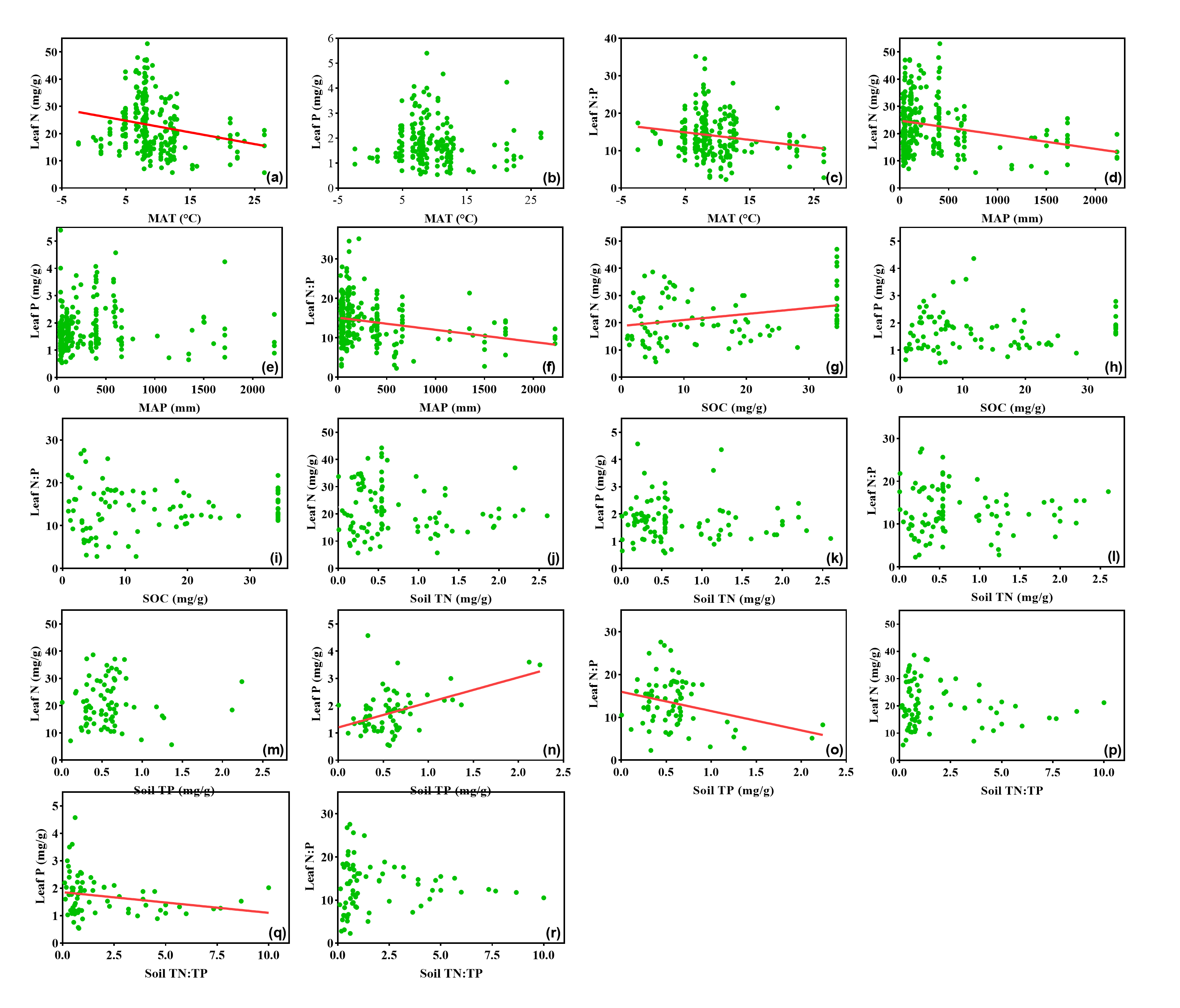


**Figure S3**

**
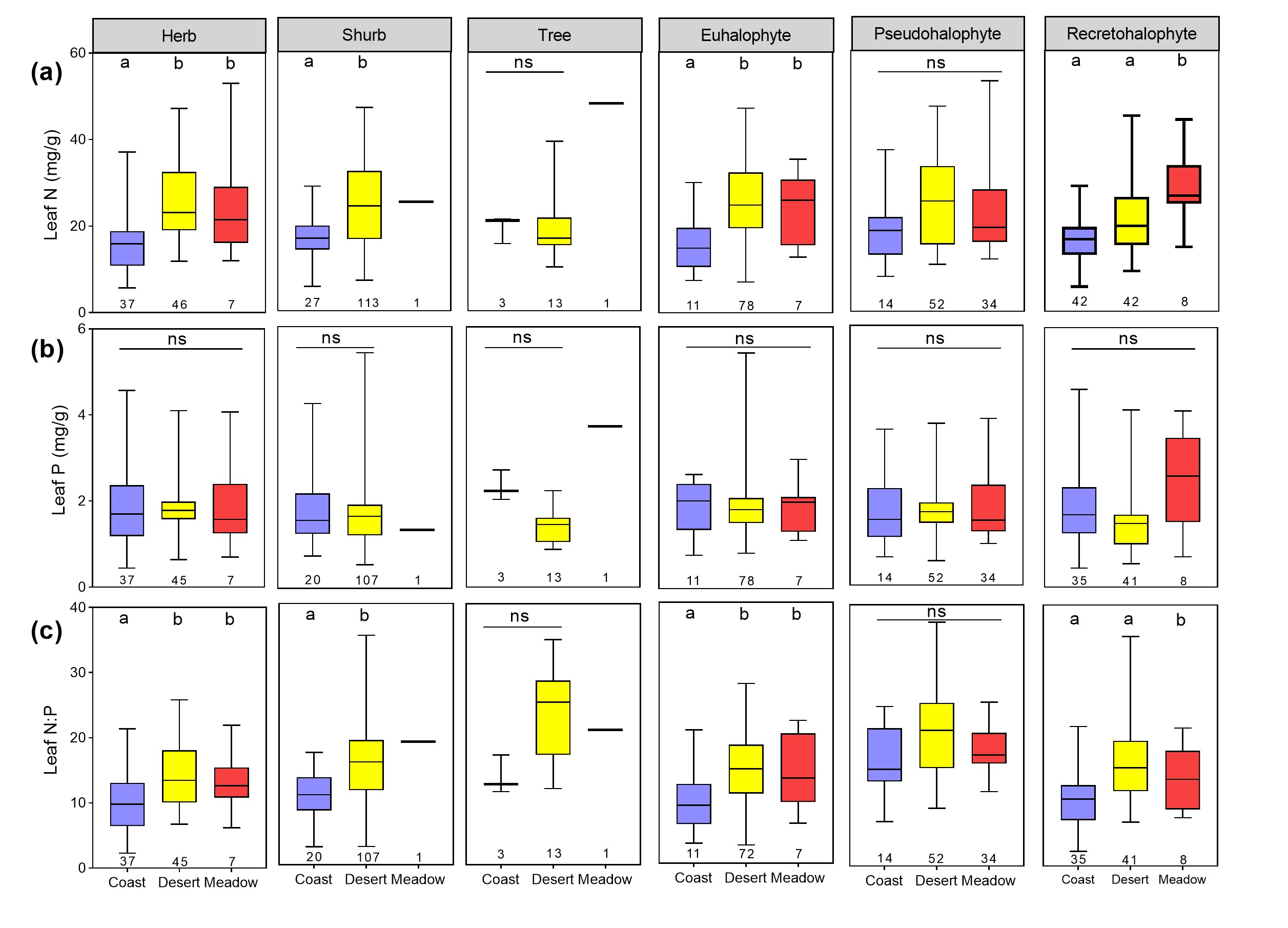
**

**Figure S4**


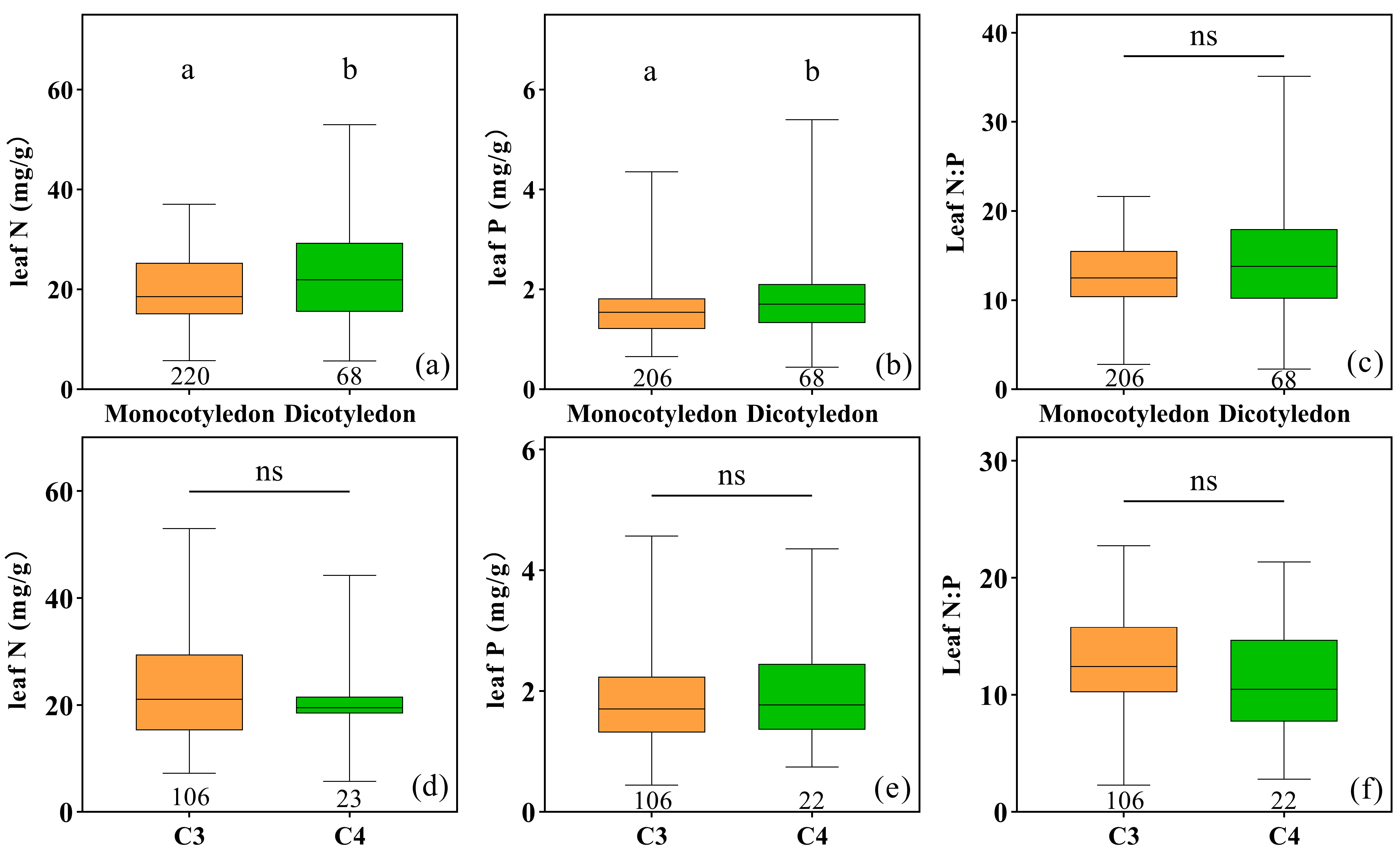


**Figure S5**


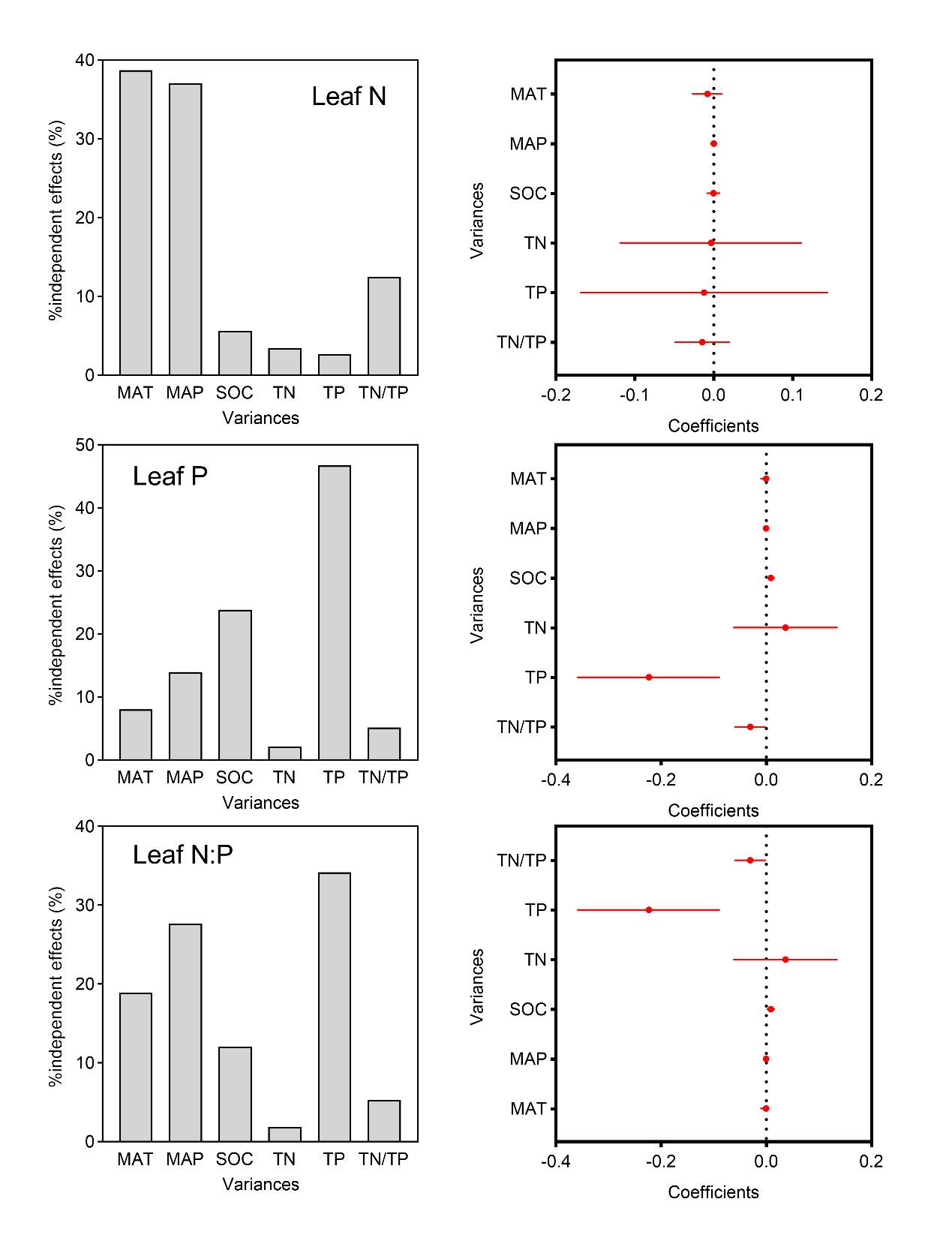


**Figure S6**
